# Complex Breakpoints and Template Switching Associated with Non-canonical Termination of Homologous Recombination in Mammalian Cells

**DOI:** 10.1371/journal.pgen.1006410

**Published:** 2016-11-10

**Authors:** Andrea J. Hartlerode, Nicholas A. Willis, Anbazhagan Rajendran, John P. Manis, Ralph Scully

**Affiliations:** 1 Department of Medicine, Beth Israel Deaconess Medical Center and Harvard Medical School, Boston, Massachusetts, United States of America; 2 Department of Pathology, Boston Children’s Hospital and Harvard Medical School, Boston, Massachusetts, United States of America; The University of North Carolina at Chapel Hill, UNITED STATES

## Abstract

A proportion of homologous recombination (HR) events in mammalian cells resolve by “long tract” gene conversion, reflecting copying of several kilobases from the donor sister chromatid prior to termination. Cells lacking the major hereditary breast/ovarian cancer predisposition genes, *BRCA1* or *BRCA2*, or certain other HR-defective cells, reveal a bias in favor of long tract gene conversion, suggesting that this aberrant HR outcome might be connected with genomic instability. If termination of gene conversion occurs in regions lacking homology with the second end of the break, the normal mechanism of HR termination by annealing (i.e., homologous pairing) is not available and termination must occur by as yet poorly defined non-canonical mechanisms. Here we use a previously described HR reporter to analyze mechanisms of non-canonical termination of long tract gene conversion in mammalian cells. We find that non-canonical HR termination can occur in the absence of the classical non-homologous end joining gene *XRCC4*. We observe obligatory use of microhomology (MH)-mediated end joining and/or nucleotide addition during rejoining with the second end of the break. Notably, non-canonical HR termination is associated with complex breakpoints. We identify roles for homology-mediated template switching and, potentially, MH-mediated template switching/microhomology-mediated break-induced replication, in the formation of complex breakpoints at sites of non-canonical HR termination. This work identifies non-canonical HR termination as a potential contributor to genomic instability and to the formation of complex breakpoints in cancer.

## Author Summary

Complex breakpoints are a recognized feature of cancer genome rearrangements, but the mechanisms that lead to their formation are undefined. Although homologous recombination (HR) is considered a potentially error-free pathway, cells lacking critical HR genes, such as the major hereditary breast/ovarian cancer predisposition genes, *BRCA1* or *BRCA2*, frequently engage error-prone homologous recombination mechanisms in which HR termination does not occur in a timely fashion. We show here that aberrant termination of HR in mammalian cells involves the use of error-prone alternative end joining mechanisms and can lead to the formation of complex breakpoints by means of template switching mechanisms. This suggests that defective termination of homologous recombination underlies some of the complex breakpoints observed in cancer cells.

## Introduction

Double strand breaks (DSBs) are dangerous lesions, the misrepair of which can contribute to genomic instability and cancer predisposition, premature aging and immunological deficiency in mammals [1–3]. A major trigger to chromosome breakage occurs during attempted replication across a damaged DNA template [4–8]. Such replication-associated DSBs may be repaired by sister chromatid recombination (SCR)—a potentially error-free pathway of homologous recombination (HR) in which the broken chromosome uses the neighboring sister chromatid as a template for repair [9–12]. Germ line mutation of HR genes contributes to hereditary breast/ovarian cancer susceptibility, Fanconi anemia and other cancer-prone or developmental disorders [1, 13–15]. Other recognized DSB repair pathways include classical non-homologous end joining (C-NHEJ), alternative end-joining (A-EJ, i.e., end-joining in the absence of one or more C-NHEJ genes) and single strand annealing (SSA) [2]. A-EJ is characterized by the dominant use of microhomology (MH)-mediated end joining (MMEJ)—rejoining events in which the two DNA ends share short stretches of homology at the breakpoint [16, 17].

Cancer genomes commonly reveal complex patterns of chromosomal rearrangement. This complexity may take the form of multiple breakpoints at the site of a chromosome rearrangement with insertion of short stretches of DNA sequence derived from ectopic loci [18–20]. The breakpoints of cancer rearrangements frequently reveal MH, but homeologous breakpoints (i.e., breakpoints with extensive but imperfect homology) and breakpoints with untemplated nucleotide addition (N-addition) are also observed [18]. Such complex rearrangements could entail rejoining of simultaneously arising chromosome breaks, break-induced copying from ectopic templates, or both [21].

A major pathway of HR repair in somatic cells is “Synthesis-dependent strand annealing” (SDSA) [22]. SDSA entails DNA end resection, loading of the Rad51 recombinase onto single stranded (ss)DNA and Rad51-mediated homologous invasion of the donor DNA molecule, such as the neighboring sister chromatid, by one of the two DNA ends. Extension of the invading/nascent strand by repair synthesis is followed by its release (“displacement”) and termination of SDSA normally occurs by annealing (i.e., homologous pairing) of the displaced nascent strand with complementary ssDNA sequences on the resected second end of the DSB. The majority of HR events triggered by a DSB resolve by “short tract” gene conversion (STGC), which typically entails repair synthesis of <100 base pairs from the donor [23–25]. A proportion of HR events resolve as “long tract” gene conversions (LTGC), in which several kilobases (up to ~10 kb) of the neighboring, undamaged sister chromatid are copied into the break site of the damaged chromosome [26, 27]. LTGC and crossing over can produce similar rearrangements in the context of an HR reporter. Where studied, these outcomes have proven to be mediated by LTGC and not by crossing over [26, 28–30]. Genetic inactivation of the major hereditary breast/ovarian cancer predisposition HR genes *BRCA1* or *BRCA2*, or of other HR genes such as the Rad51 paralogs *Rad51C*, *XRCC2* or *XRCC3* biases HR in favor of LTGC [28–34]. Thus, understanding the mechanisms underlying LTGC in mammalian cells may yield insight into mechanisms of genomic instability in HR-defective hereditary breast/ovarian cancer-predisposition syndromes.

Very long gene conversions in *Saccharomyces cerevisiae* are mediated by break-induced replication (BIR), which can copy >100 kilobases from the donor molecule [35–37]. The BIR copying mechanism in *S*. *cerevisiae* is conservative, rather than the semi-conservative mechanism of a conventional replication fork [38, 39]. BIR in *S*. *cerevisiae* is dependent on the Pif1 helicase and entails a migrating bubble mechanism [39, 40]. Gene conversions in *S*. *cerevisiae* that ultimately resolve as BIR may reveal homologous template switches during the early stages of the process, suggesting that the initial steps of BIR can be mediated by less robust copying mechanisms [41]. Further, spontaneous somatic gene conversions in *S*. *cerevisiae* reveal a bimodal distribution of tract lengths, with median peaks at 6 kb and >50 kb [42]. Taken together, these studies suggest that classical BIR and LTGC, although topologically similar processes, retain some mechanistic differences.

If the site of HR termination lacks homology with the second (non-invading) end of the DSB, the classical SDSA mechanism of termination by annealing with the resected second end of the DSB is not available. Under these circumstances, HR termination may be mediated by end joining mechanisms [26, 27, 43, 44]. Breakpoints of non-canonical HR termination often reveal MH, suggesting a role for A-EJ in this process [43, 44]. However, the genetic regulation of non-canonical HR termination in mammalian cells is currently undefined. In *Drosophila melanogaster*, non-homologous termination of HR repair of a transposase-induced break is independent of the C-NHEJ gene *LIG4* and is mediated by the error-prone DNA polymerase PolΘ, encoded by the *POLQ* gene [45, 46]. Here, we use a previously described mammalian reporter of LTGC between sister chromatids [27] to analyze mechanisms of non-canonical LTGC termination in *XRCC4* conditional and isogenic *XRCC4* null mouse embryonic stem (ES) cells [47, 48]. Our work reveals that non-canonical termination of HR in mammalian cells is independent of *XRCC4* and can lead to the formation of complex breakpoints, mediated by template switching. This suggests that non-canonical termination of HR may contribute to the formation of complex breakpoints in the cancer genome.

## Results

### Non-canonical termination of mammalian HR does not require *XRCC4*

**Fig 1:**
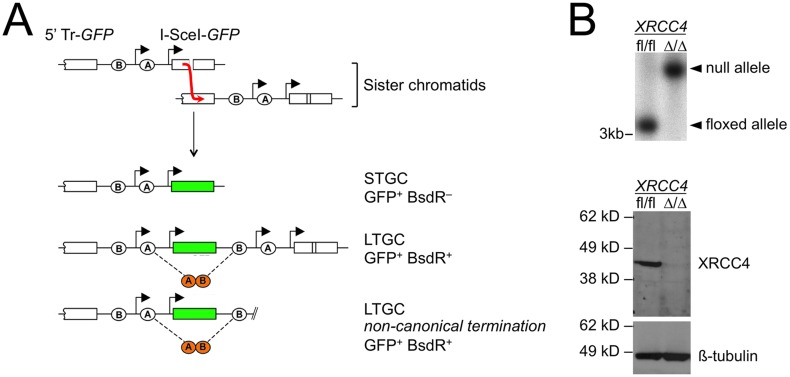
Method for identifying non-canonical HR termination products in mammalian cells. (A) Schematic of the HR reporter. Duplication of a blasticidin resistance cassette during LTGC allows expression of wt *BsdR* by splicing. Thus, I-SceI-induced STGCs are GFP^+^ and Bsd sensitive (BsdR^–^), while I-SceI-induced LTGCs are GFP^+^ and Bsd resistant (BsdR^+^). Most LTGCs resolve as “*GFP* triplication” events, but a small fraction of LTGCs resolve by non-canonical mechanisms. Non-canonical LTGC termination products can be distinguished by the structure of the LTGC product, as shown. (B) Characterization of *XRCC4*^fl/fl^ and *XRCC4*^Δ/Δ^ Cre-treated HR reporter clones. Upper panel: Southern blotting, as described in Materials and Methods. Lower panel: western blotting for XRCC4 or for ß-tubulin loading control.

We previously described a HR reporter that enables positive selection of both short tract (STGC) and long tract gene conversions (LTGC) between sister chromatids in response to a site-specific DSB induced by the rare-cutting homing endonuclease I-SceI (Fig 1) [27]. Briefly, we positioned two artificial exons of the gene encoding blasticidin S deaminase (here termed “*BsdR*”) in a non-productive orientation between the two *GFP* copies of an HR reporter. Parental cells, or products of STGC, remain blasticidin sensitive (*BsdR*^–^; Fig 1A). In contrast, LTGC duplicates the *BsdR* cassette, thereby allowing expression of wild type (wt) *BsdR* by splicing (Fig 1A). LTGC is experimentally defined here as a gene conversion of >1.03kb—sufficient to duplicate exon B of the blasticidin cassette.

The most abundant I-SceI-induced HR product is STGC, in which the broken copy of *GFP* is converted to wild type *GFP*, leaving the reporter structure otherwise unchanged (Fig 1A). In wild type cells, approximately 5% of all I-SceI-induced GFP^+^ products resolve by LTGC [28, 29, 47, 49]. LTGC frequently results in triplication of the *GFP* copies within the repaired sister chromatid (Fig 1A). However, a small proportion of I-SceI-induced LTGCs are terminated in regions lacking homology with the second end of the DSB [26, 27, 29]. These LTGCs must be terminated by non-canonical mechanisms (Fig 1A).

To study the contribution of C-NHEJ to non-canonical HR termination, we introduced the above-noted “long tract” HR/SCR reporter into mouse embryonic stem (ES) cells carrying biallelic conditional (“floxed”) alleles of *XRCC4* (*XRCC4*^fl/fl^ ES cells) [48, 50]. We identified individual clones in which a single, intact copy of the reporter had been integrated into the *ROSA26* locus, as described previously and in Materials and Methods [49]. We transduced two distinct *XRCC4*^fl/fl^ HR/SCR reporter ES cell clones with adenovirus encoding the Cre recombinase and screened Cre-treated cells for derivative clones that either had or had not undergone biallelic Cre-mediated deletion of *XRCC4*. Southern and western blotting identified *XRCC4*^Δ/Δ^ and *XRCC4*^fl/fl^ derivatives of these cells (examples in Fig 1B). We transfected *XRCC4*^fl/fl^ and, in parallel, *XRCC4*^Δ/Δ^ HR/SCR reporter ES cells with I-SceI (with appropriate controls as described in Materials and Methods), and scored HR products as the frequency of I-SceI-induced GFP^+^ and *BsdR*^+^ events (LTGCs). The ratio LTGC:Total HR (BsdR^+^ GFP^+^: Total GFP^+^) is a measure of the probability that a given HR event will resolve as LTGC. This value was ~3% in each cell type, suggesting that *XRCC4* does not directly influence the probability of engaging LTGC during I-SceI-induced HR.

We amplified I-SceI-induced BsdR^+^ colonies from two *XRCC4*^fl/fl^ HR/SCR reporter clones (n = 163) and two isogenic *XRCC4*^Δ/Δ^ HR/SCR reporter clones (n = 211), prepared genomic DNA (gDNA), and analyzed the underlying structure of the LTGC product by Southern blotting, as described in Materials and Methods—results summarized in Table 1. We noted examples of non-canonical LTGC termination in both *XRCC4*^fl/fl^ (6/163; 3.7%) and *XRCC4*^Δ/Δ^ (5/211; 2.4%) HR/SCR reporter cells (difference not significant by Fisher’s exact test). This establishes that non-canonical HR termination can occur in the absence of the C-NHEJ gene *XRCC4*. A proportion of LTGCs produced aberrant Southern blot patterns, either in the form of off-size bands or additional *GFP*-hybridizing bands, which defied easy interpretation. 14/211 (6.6%) of all LTGCs examined in *XRCC4*^Δ/Δ^ HR/SCR reporter cells were aberrant; the equivalent proportion in *XRCC4*^fl/fl^ HR/SCR reporter cells was 2/163 (1.2%); (P = 0.0102 by Fisher’s exact test). The higher proportions of aberrant LTGCs noted in *XRCC4*^Δ/Δ^ HR/SCR reporter cells is consistent with the known role of *XRCC4* in suppressing chromosomal rearrangements [51, 52]. Analysis of one of these aberrant LTGCs in *XRCC4*^Δ/Δ^ HR/SCR reporter cells is presented below.

**Table 1:** I-SceI-induced LTGC products in *XRCC4*^fl/fl^ and *XRCC4*^Δ/Δ^ cells.

Genotype	*XRCC4*^fl/fl^	*XRCC4*^Δ/Δ^
*GFP* triplication	155	192
LTGC non-canonical termination	6	5
Aberrant	2	14

Table 1 summarizes Southern blot analysis of I-SceI-induced blasticidin-resistant clones in *XRCC4*^fl/fl^ (n = 163) and *XRCC4*^Δ/Δ^ (n = 211) SCR reporter cells. Fisher’s exact test *XRCC4*^fl/fl^ *vs*. *XRCC4*^Δ/Δ^ for *GFP* triplication *vs*. non-canonically terminated LTGC: not significant. Fisher’s exact test *XRCC4*^fl/fl^ *vs*. *XRCC4*^Δ/Δ^ for *GFP* triplication *vs*. aberrant LTGCs (excludes non-canonically terminated LTGC products): P = 0.0102.

### Microhomology-mediated end joining mediates non-canonical LTGC termination

**Fig 2:**
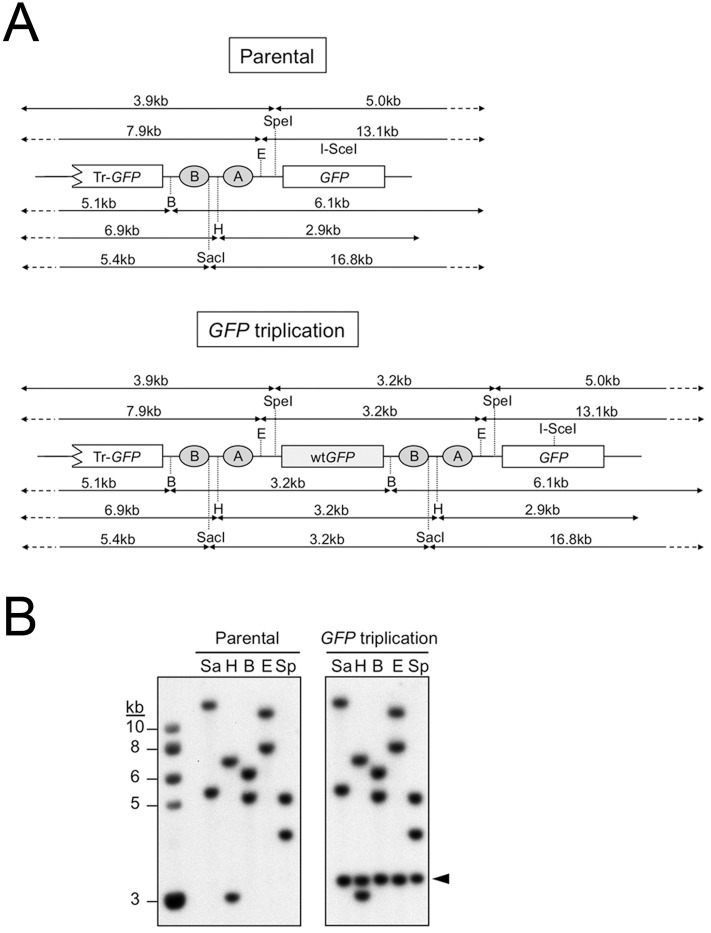
Restriction mapping of parental reporter and of LTGC “*GFP* triplication” products. (A) Expected *GFP*-hybridizing gDNA restriction fragment sizes for HR reporter at the *ROSA26* locus. Upper panel: parental reporter; lower panel: “*GFP* triplication” outcome of LTGC. *GFP* copies within the reporter are shown. Filled ovals: artificial *BsdR* exons A and B. Restriction enzyme sites shown are SpeI (Sp), EcoRI (E), BamHI (B), HindIII (H) and SacI (Sa). Note that each of these restriction endonucleases, which cut target sites between the two *GFP* copies within the parental reporter, generate an additional 3.2kb *GFP*-hybridizing band in the context of the “*GFP* triplication” outcome. (B) Genomic DNA from parental and “*GFP* triplication” LTGC clones, as shown, was digested with the restriction enzymes shown (code as described above) and analyzed by Southern blotting (*GFP* probe). The 3.2kb amplification product characteristic of the “*GFP* triplication” LTGC outcome is marked with an arrowhead.

**Fig 3:**
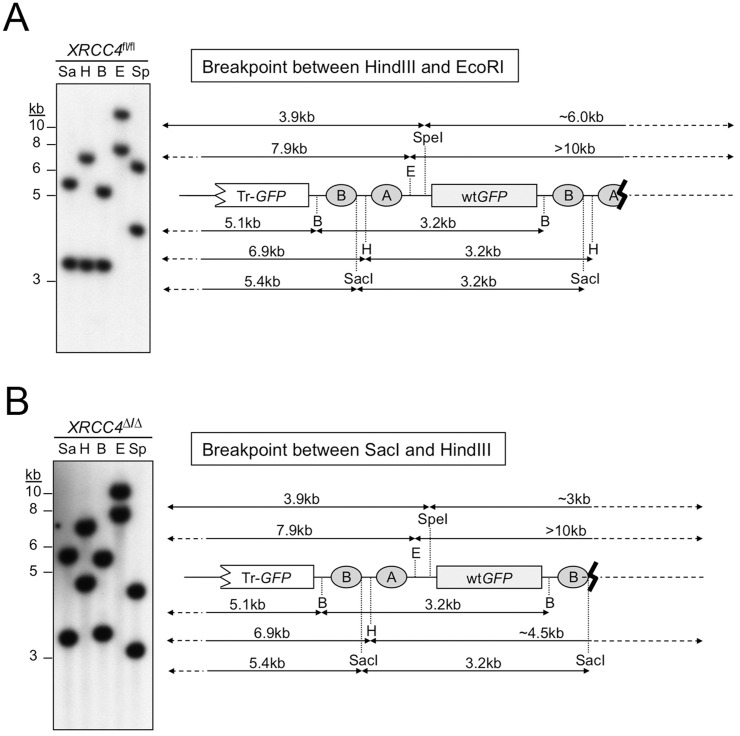
Restriction mapping of products of non-canonical LTGC termination. Genomic DNA from two clones in which LTGC was terminated by non-canonical mechanisms was digested with the restriction enzymes shown and analyzed by Southern blotting (*GFP* probe). Restriction enzymes used were SacI (Sa), HindIII (H), BamHI (B), EcoRI (E) and SpeI (Sp). Cartoons on right show restriction fragment sizes observed for HR reporter at the *ROSA26* locus. The presence or absence of the 3.2kb amplification product in each restriction digest helps to localize the site of LTGC termination within the reporter. (A) *XRCC4*^fl/fl^ clone in which termination of LTGC occurred between HindIII and EcoRI sites within the HR reporter. EcoRI and SpeI digests lack the 3.2kb amplification product. (B) *XRCC4*^Δ/Δ^ clone in which termination of LTGC occurred between SacI and HindIII sites within the HR reporter. HindIII, EcoRI and SpeI digests lack the 3.2kb amplification product. In this clone, the right hand arms of the SpeI and HindIII digests are much smaller (SpeI) or larger (HindIII) than would be predicted. This is explained by the deletion of ~3.5kb from the second end of the DSB, as revealed by sequencing (see Fig 6B).

**Fig 4:**
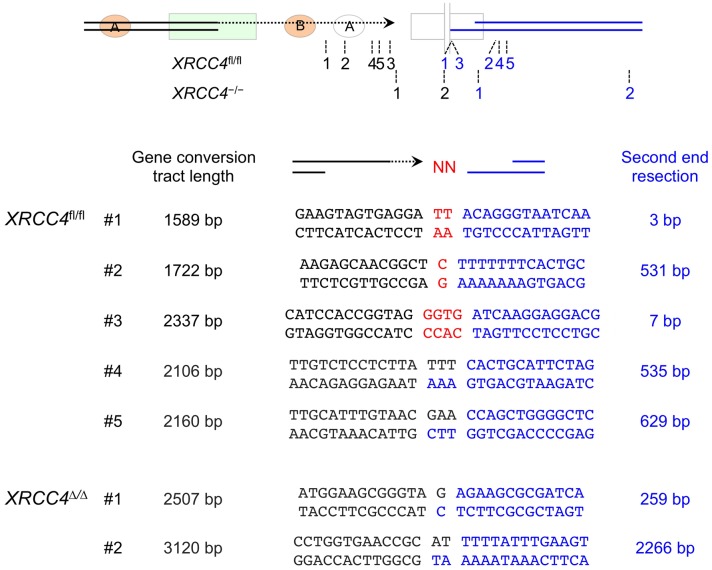
Breakpoints of non-canonical LTGC termination in five *XRCC4*^fl/fl^ and two *XRCC4*^Δ/Δ^ clones. Cartoon shows approximate positions of breakpoints. Black numbers mark site of LTGC termination; paired blue numbers mark extent of second end resection for the same clone (not to scale). Numbers correlate with the numbered clones in lower panel, showing length of gene conversion tract (black) and extent of second end resection (blue) in each clone, with genotype as indicated. Red nucleotides: N-insertions at the breakpoint. Dual black/blue nucleotide sequences at the breakpoint represent microhomology.

The unrearranged parental reporter and the major “*GFP* triplication” LTGC product produce predictable patterns of hybridization following gDNA digestion with a panel of restriction endonucleases (Fig 2). We made the assumption that non-canonical termination of LTGC normally entails rejoining with the second end of the DSB and used the specific pattern of Southern blot hybridizations to deduce the likely site of non-canonical LTGC termination in *XRCC4*^fl/fl^ or *XRCC4*^Δ/Δ^ LTGC clones. Two such examples are shown in Fig 3. We were able to clone the breakpoints of six *XRCC4*^fl/fl^ and three *XRCC4*^Δ/Δ^ non-canonical LTGC termination products (see Materials and Methods). The cloned breakpoints did indeed reflect rejoining to the second end of the DSB, which had undergone varying degrees of resection (Fig 4). Each breakpoint revealed use of MMEJ or untemplated nucleotide addition (N-addition) at the breakpoint. It has been suggested that N-addition breakpoints of the type observed here might also be products of MMEJ-type rejoining [45]. There were no blunt-ended non-homologous breakpoints in this limited sample and no breakpoints were suggestive of dual homologous invasions by both ends of the original I-SceI-induced DSB. Thus, non-canonical termination of HR can occur in the absence of the C-NHEJ gene *XRCC4* and entails use of MMEJ/N-addition rejoining mechanisms, implicating A-EJ as a contributing mechanism.

### Analysis of an aberrant LTGC product of *XRCC4*^Δ/Δ^ HR/SCR reporter cells

**Fig 5:**
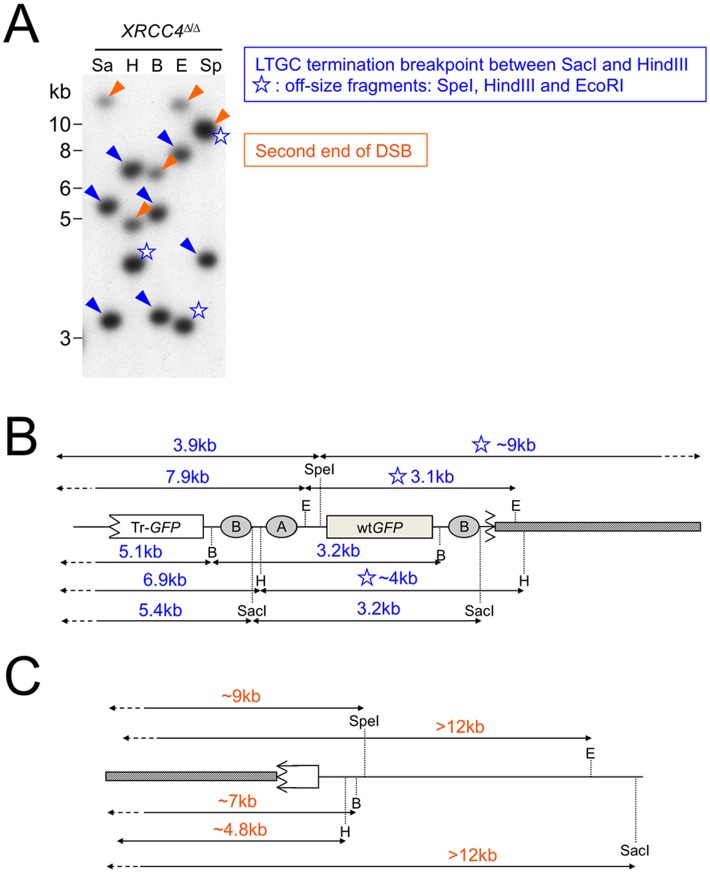
Restriction mapping of an aberrant LTGC rearrangement in *XRCC4*^Δ/Δ^ HR/SCR reporter cells. (A) Restriction analysis of aberrant LTGC product in *XRCC4*^Δ/Δ^ HR/SCR reporter cells. Genomic DNA was digested with the restriction enzymes shown and analyzed by Southern blotting (*GFP* probe). Restriction enzymes used were SacI (Sa), HindIII (H), BamHI (B), EcoRI (E) and SpeI (Sp). Patterns suggest that LTGC was terminated by non-canonical mechanisms. Blue arrow-heads: deduced products of non-canonical LTGC termination. Blue star: off-size restriction fragments of HindIII, EcoRI and SpeI digests are inconsistent with rejoining with the second end of the original I-SceI-induced DSB (compare with Fig 2). Orange arrow-heads: fainter bands may represent the half-copy of *GFP* retained by the second end of the original I-SceI-induced DSB. Note that the upper band of SpeI-restricted gDNA has a greater intensity than other bands, suggesting presence of two distinct co-migrating *GFP*-hybridizing fragments, or a single fragment containing >1 copy of *GFP*. (B) Deduced rearrangement of the non-canonically-terminated LTGC. Blue star: off-size restriction fragments of HindIII, EcoRI and SpeI digests. Note that each of these off-size fragments spans the predicted breakpoint of LTGC termination. This suggests that this LTGC event terminated by rejoining to ectopic chromosomal sequences (grey bar in the figure). (C) Deduced rearrangement of the second end of the DSB. Note that *GFP*-hybridizing fragments of SpeI, BamHI and HindIII restriction digest are off-size, potentially consistent with rejoining of the second end of the DSB with ectopic chromosomal sequences (grey bar).

We used a similar restriction mapping approach to analyze one aberrant LTGC product identified in *XRCC4*^Δ/Δ^ HR/SCR reporter cells. As discussed above, aberrant LTGC products characteristically reveal off-size or additional *GFP*-hybridizing bands by Southern blotting. One such aberrant clone is shown in Fig 5. Southern analysis appeared to show two groups of *GFP*-hybridizing bands with distinct intensities. Importantly, these groups were not separated by recloning of the cells, indicating that all the *GFP* fragments visualized by Southern blotting reside within one nucleus. We interpret the Southern blot pattern as a case of non-canonical LTGC termination (blue arrow-heads Fig 5) in which LTGC termination occurred between the SacI and HindIII sites within the reporter. However, all restriction fragments involving enzymes beyond the SacI site (i.e., HindIII, EcoRI and SpeI) reveal off-size *GFP*-hybridizing bands (Fig 5B). These fragments do not match restriction fragment patterns of *ROSA26* sequence up to 50 kb beyond the second end of the DSB. This suggests that LTGC termination in this case entailed incorporation of ectopic chromosomal sequences. We interpret the fainter *GFP*-hybridizing bands in this Southern blot (orange arrow-heads) as possible products of the second end of the break (Fig 5C). If so, the rearrangement underlying this aberrant LTGC product could entail a gross chromosomal rearrangement (GCR) initiated by non-canonical LTGC termination. Alternatively, the ectopic sequences (grey bars) depicted in Fig 5A and 5B might be part of one single insertion of several kilobases between the site of LTGC termination and the second end of the break. In this regard, the solitary ~9 kb SpeI fragment in Fig 5A, which appears to have a higher intensity than all other bands, could potentially span this insertion, while retaining *GFP* sequences from both sides of the termination breakpoint. However, our attempts to amplify such a putative insertion product between the two ends of the break have not yet been successful. The notion that non-canonical LTGC termination might lead to GCR is consistent with the expected greater availability of free DNA ends in *XRCC4*^Δ/Δ^ cells, where efficient C-NHEJ mechanisms are compromised. This clone is an example of non-canonical LTGC termination that presents with an aberrant LTGC pattern by Southern blotting. However, until this and other aberrant LTGC products are mapped and sequenced, it would not be valid to conclude that all aberrant LTGC outcomes arise from non-canonical LTGC termination.

### Complex breakpoints associated with non-canonical termination of LTGC

**Fig 6:**
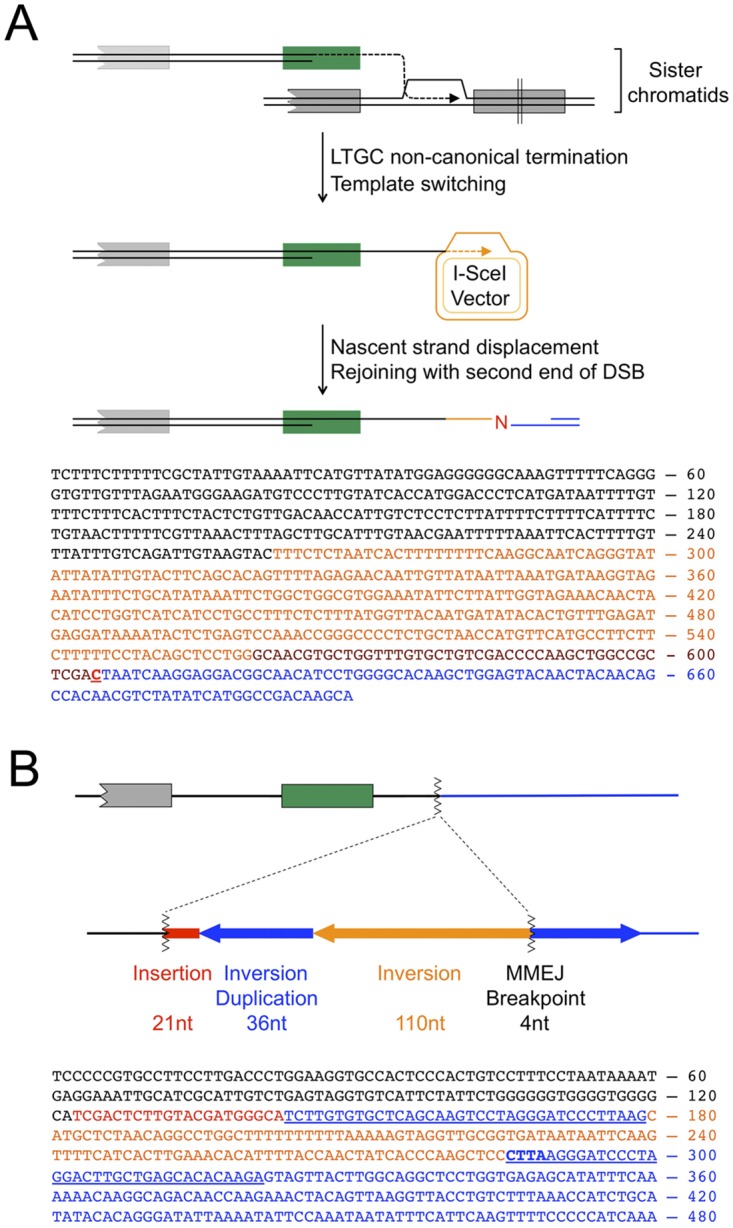
Template switching and complex breakpoints during non-canonical LTGC termination. (A) Homologous template switching during non-canonical LTGC termination in a *XRCC4*^fl/fl^ clone. **Upper panel:** Cartoon depicts the HR-mediated template switch between the displaced nascent strand product of LTGC (black) and identical rabbit β-globin intron sequences within the episomal I-SceI expression vector. **Lower panel:** Sequence of the homologous template switch complex breakpoint. Orange: intron sequences copied from the I-SceI expression vector. Brown: additional sequence copied from the episomal I-SceI expression vector. Red: single N-addition at second breakpoint. Blue: second end of the DSB, resected 9 bp prior to end joining with the twice-displaced nascent strand. (B) Microhomology-mediated complex breakpoint formation during non-canonical LTGC termination in a *XRCC4*^Δ/Δ^ clone. Southern blotting analysis of this clone is presented in Fig 3B. **Upper panel:** Cartoon shows map of the complex breakpoint, which involved rearrangement of the second (non-invading) DNA end. Red: 21nt insertion. Blue arrows: duplicated 36bp sequence from second end of DSB (located 3579-3614bp from the I-SceI site). Orange arrow: Inverted 110bp sequence adjacent to duplicated sequence (located 3469-3578bp from the I-SceI site). The MMEJ breakpoint within the second DNA end is located 3579-3582bp downstream of the I-SceI site. Blue sequences (including correctly oriented blue arrow) to the right of MH breakpoint are unrearranged *ROSA26* locus. With the exception of the inverted 110 bp sequence, a segment of the second DNA end ~3.5kb adjacent to the I-SceI site was deleted during the rearrangement. **Lower panel:** Sequence of the MH-mediated complex breakpoint. Black: LTGC product (gene conversion tract length was 1249bp). Red: 21nt insertion. First blue underlined: inverted 36bp repeat. Orange: 110bp inversion. Second blue underlined: correctly oriented 36bp repeat, contiguous with unrearranged *ROSA26* sequence. Bold underlined blue: 4bp MH breakpoint. Hypothetical model of this complex breakpoint is presented in Fig 7.

In one *XRCC4*^fl/fl^ clone in which LTGC had been terminated by non-canonical mechanisms, sequencing revealed two distinct breakpoints: one homologous and one N-addition breakpoint. The homologous breakpoint reflected incorporation of sequences from the episomal I-SceI expression vector within the repaired sister chromatid (Fig 6A). The vector sequence had been incorporated at a site of perfect and extensive homology between the chromosomally integrated HR/SCR reporter and the episomal plasmid, based upon shared rabbit β-globin intron sequences [27, 53]. Following LTGC using the sister chromatid as template, a template switching mechanism allowed the displaced nascent strand to invade homologous sequences on the episomal plasmid. After further nascent strand synthesis of ≥342 bp (the exact point of homologous invasion of the episomal plasmid is not definable), the newly extended nascent strand was displaced from the plasmid template and was joined to the second end of the I-SceI-induced chromosomal break, with insertion of one nucleotide at this second (non-homologous) breakpoint (Fig 6A). Thus, non-canonical termination of LTGC can entail homologous template switching—a phenomenon known to be associated with LTGC and BIR in *S*. *cerevisiae* [41, 54].

**Fig 7:**
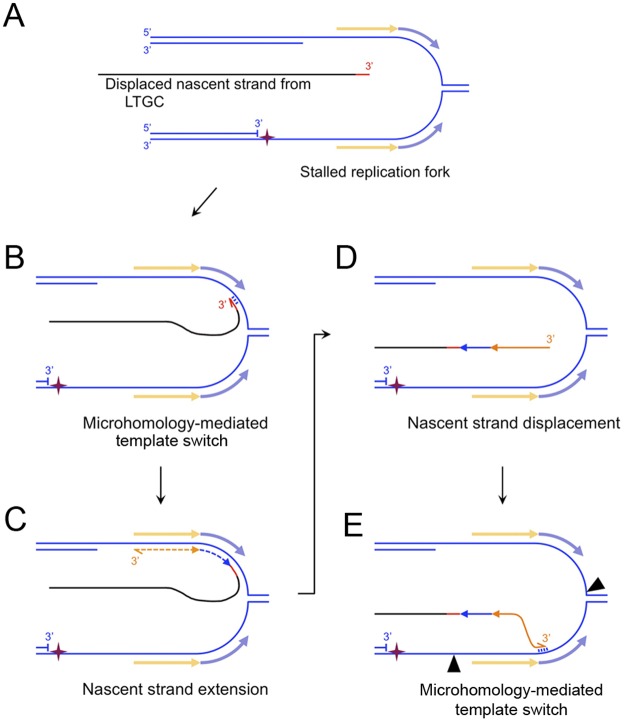
MMBIR model of complex breakpoint shown in Fig 5B. Strand separation occurs within the DNA of the second end of the break ~3.5 kb from the I-SceI site. One possible source depicted here is a stalled replication fork. The pale orange and blue arrows flanking the stalled fork represent the exposed ssDNA sequences that template the inversion (orange) and inversion-duplication (blue) sequences identified within the LTGC breakpoint (A) The displaced nascent strand product of LTGC (black) acquires a ≥21bp insertion (red; whether templated or untemplated is unknown). (B) Microhomology-mediated base-pairing between the 3’ end of the displaced nascent strand and ssDNA of the stalled replication fork. (C) The lagging strand template enables retrograde nascent strand extension (“MMBIR”), generating the inversion sequences as shown. (D) Displacement of the nascent strand. (E) Four base pair MH-mediated (Fig 6B) annealing of the 3’ end of the displaced nascent strand with the 5’ end of the duplicated region on the leading strand. Black arrowheads: sites of endonucleolytic cleavage that would enable completion of rearrangement by MMEJ-mediated rejoining. Alternatively, more extensive MMBIR copying could complete the rearrangement.

A second complex breakpoint of non-canonical LTGC termination was present in one *XRCC4*^*Δ/Δ*^ clone. Sequencing of the breakpoint revealed an inversion/duplication rearrangement of the second end of the DSB (Fig 6B; Southern blot analysis of this clone is shown in Fig 3B), involving at least two breakpoints in close proximity to one another. The first breakpoint entailed a 21bp insertion at the site of non-canonical LTGC termination, showing 16bp identity with several heterologous loci in the mouse genome (if templated, this 21bp insertion could represent two independent breakpoints). The second was a 4bp MH breakpoint generated during ligation to the second end of the DSB, with an accompanying complex deletion/inversion/duplication rearrangement of the second end of the DSB. Although the mechanisms underlying this complex rearrangement are a matter of speculation, the rearrangement suggests that the nascent strand, having been displaced from the donor sister chromatid during LTGC termination, underwent further rounds of MH-mediated template switches and short nascent strand extension—a process termed “microhomology-mediated BIR” (MMBIR) [55]. Fig 7 depicts how this MMBIR rearrangement could have arisen through a fork stalling and template switching (FoSTeS) mechanism [56]. Notably, the 146 bp inversion fragment (Fig 6B) is of a size consistent with FoSTeS-type copying from a lagging strand donor.

## Discussion

We used the positive selective power of a HR/SCR reporter to capture rare LTGCs in which HR had been terminated by non-canonical mechanisms in *XRCC4*^fl/fl^ and *XRCC4*^Δ/Δ^ mouse ES cells. Rejoining with the second end of the chromosomal break entails use of *XRCC4*-independent MMEJ (i.e. A-EJ), in agreement with previous studies in *D*. *melanogaster* [45, 46]. A notable finding of the current study is that non-canonical HR termination in mammalian cells may entail homologous template switching or MH-mediated template switching (i.e., MMBIR) prior to rejoining with the second DNA end, leading to the formation of complex breakpoints at the site of HR termination. Long gene conversions during gap repair in *D*. *melanogaster* have been proposed to entail cycles of invasion and displacement of the nascent strand, with an implied potential for template switching [57]. Both homologous template switches and MMBIR have been described in *S*. *cerevisiae* during LTGC/BIR, suggesting that these error-prone mechanisms of HR termination are evolutionarily conserved [41, 54, 58]. Our findings provide direct evidence of homologous template switching during mammalian HR, highlighting the extreme reactivity of the displaced nascent strand and its potential significance as an instigator of genomic instability. Given the likely importance of template switching mechanisms in the formation of complex breakpoints in cancer cells, our findings suggest that aberrant HR termination may underlie some of the complex breakpoints observed in cancer genomes [18–21].

A striking feature of the breakpoints associated with non-canonical LTGC termination is the frequent use of MMEJ/insertional rejoining mechanisms. The channeling of repair into an MMEJ mechanism is likely best explained by the DNA structures that are presented for rejoining. Both the displaced nascent strand and the resected second end of the break possess extended 3’ ssDNA tails. These are poor substrates for Ku binding and, hence, for C-NHEJ-mediated rejoining, leading to a preference for A-EJ [59]. Completion of non-canonical LTGC by MMEJ-mediated rejoining to the second end of the DSB may suppress more deleterious outcomes, such as template switching, BIR and chromosome translocation, at sites of non-canonical HR termination. Direct testing of this hypothesis must await the development of more readily quantifiable systems for studying non-canonical HR termination in mammalian cells. However, this idea is strongly corroborated by work on the A-EJ mediator PolΘ, which suppresses genomic instability in mammalian cells and prevents large deletions at sites of replication arrest or at transposase-induced gaps in model organisms [46, 60–64]. Conversely, unrestrained LTGC in *BRCA* mutant and other HR-defective cells might channel HR towards these deleterious outcomes as a mechanism of genomic instability in tumorigenesis [28–30].

In the cell lines studied here, non-canonical LTGC termination accounts for ~3% of all LTGCs in *XRCC4*^fl/fl^ cells, corresponding to ~0.1% of all measured GFP^+^ I-SceI-induced HR events. These low frequencies may nonetheless be highly significant for genomic instability and cancer predisposition, since cancer initiation and progression result from stochastic events on a “per cell” basis. The significance of non-canonical termination of LTGC may be greater than is suggested by the above calculations, since the repetitive structure of the HR reporter used here presents two opportunities for HR termination by annealing: during STGC and in the termination of LTGC by “*GFP* triplication” (Fig 1). In contrast, when gene conversion occurs within non-repetitive sequences, STGC alone provides an opportunity for HR to be terminated by annealing. In this more natural setting, presumably all LTGCs must resolve either by non-canonical termination mechanisms or by BIR. In this regard, it is relevant that mammalian cells lacking the major hereditary breast/ovarian cancer predisposition genes *BRCA1* or *BRCA2* or other cancer predisposition HR genes reveal a bias towards LTGC [28, 31–34]. This bias is even more marked at stalled replication forks, where >80% of HR events may resolve as LTGCs in *BRCA*/HR-defective cells [30]. In this setting, the arrival of a converging replication fork and the activity of stalled fork endonucleases may be additional determinants of genomic instability [65]. The work described here identifies mechanisms by which dysregulated LTGC may contribute to genomic instability in *BRCA*/HR-defective cells and in general tumorigenesis.

## Materials and Methods

*Plasmids—*The sister chromatid recombination reporter was previously characterized. Expression plasmids for *I-SceI* and *GFP* were described previously [27, 49]. New constructs described here were generated by standard cloning procedures.

*Cell Lines and Cell Culture—XRCC4*^fl/fl^ mouse embryonic stem (ES) cells were obtained from Catherine Yan and Frederick Alt and have been described previously [48]. ES cells were maintained in ES medium on either irradiated MEF feeder cells or gelatinized plates. To generate SCR reporter stable lines, 20μg of KpnI-linearized SCR reporter plasmid was electroporated into 2x10^7^ *XRCC4*^fl/fl^ ES cells and cells were seeded into 60mm dishes with neomycin resistant feeder mouse embryonic fibroblasts and 400μg/mL G418 (Sigma-Aldrich) was added to the medium 1 day after electroporation. Beginning 1 week after continuous selection, G418-resistant colonies were isolated and screened by Southern blotting for single-copy SCR reporter integration. To generate isogenic *XRCC4*^fl/fl^, *XRCC4*^fl/Δ^ and *XRCC4*^Δ/Δ^ SCR cell lines, adeno-Cre infection was performed as described previously [49], followed by screening of derivative cell lines by Southern blotting.

*Recombination Assays—*1.6x10^5^ trypsinized ES cells were transfected with 0.5μg plasmid DNA using Lipofectamine 2000 (Invitrogen) in a 24-well plate. Transfection efficiency was measured by parallel transfection of wt*GFP* expression vector (at 1:10 dilution in empty vector). GFP^+^ frequencies were measured 72 hr post-treatment by flow cytometry using an FC500 (Beckman Coulter) as described previously [27]. To assay LTGC events, cells were counted and replated at 1-3x10^5^ cells per gelatinized 100mm dish in triplicate into media containing 5μg/mL blasticidin (Invitrogen). Approximately 2 weeks later, blasticidin resistant colonies were stained and counted or expanded for molecular analysis. Plating efficiency was determined by plating 3-5x10^2^ cells per gelatinized 100mm dish in triplicate into media lacking selection. HR measurements were corrected for background levels of HR events, transfection efficiency and plating efficiency, as described previously [49].

*Southern Blotting—*Genomic DNA was extracted from 5-20x10^6^ cells using the ArchivePure Cell/Tissue Kit (5 PRIME). *GFP* and *XRCC4* Southern blots were carried out as previously described [27, 47, 50, 66].

*Western Blotting—*Cell lysates were prepared using RIPA buffer (50 mM Tris-HCl [pH 8.0], 1.0% NP-40, 150 mM NaCl, 0.5% sodium deoxycholate, 0.1% SDS) containing protease inhibitors (Roche). Protein concentration was estimated using Bradford’s Reagent (Sigma-Aldrich). Cellular proteins were resolved by SDS-PAGE on NuPAGE Novex Bis-Tris Gels (Invitrogen), transferred to nitrocellulose membrane (Bio-Rad semi-dry transfer system, 40 mA overnight). The membrane was blocked with 5% nonfat milk in 0.05% PBST (0.05% Tween 20, in PBS) and incubated with rabbit polyclonal anti-XRCC4 1:200 (Sigma-Aldrich) or mouse monoclonal anti-β-tubulin 1:200 (Abcam) at room temperature for 3 hrs. Membranes were washed in 0.05% PBST, incubated with peroxidase-conjugated Protein A (GE Healthcare) or goat anti-mouse antibody (Jackson ImmunoResearch) and developed using high-sensitivity ECL (PerkinElmer).

*PCR and Sequencing—*Breakpoints were amplified using AccuPrime *Taq* DNA Polymerase High Fidelity (Invitrogen) according to manufacturers instructions. The PCR products were excised from the gel and purified using the QIAquick Gel Extraction Kit (QIAGEN) and subsequently cloned into the pGEM-T Easy vector (Promega). Sequencing was performed at the Dana-Farber/Harvard Cancer Center DNA Resource Core.
