# Correction: Complex Breakpoints and Template Switching Associated with Non-canonical Termination of Homologous Recombination in Mammalian Cells

**DOI:** 10.1371/journal.pgen.1006509

**Published:** 2016-12-13

**Authors:** Andrea J. Hartlerode, Nicholas A. Willis, Anbazhagan Rajendran, John P. Manis, Ralph Scully

There is an error in the caption for Fig 7. Specifically, the title of the caption incorrectly references Fig 5B. The correct reference should be to Fig 6B. Please see the complete, correct Fig 7 caption here.

**Fig 7:**
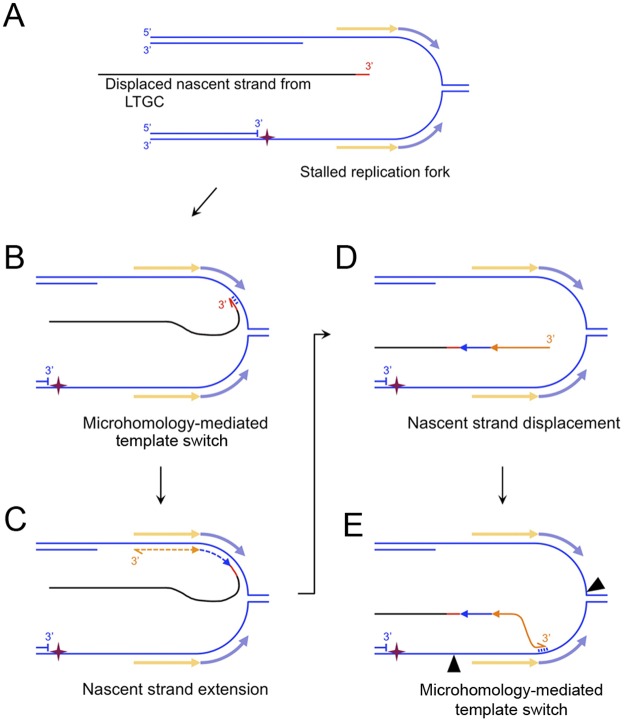
MMBIR model of complex breakpoint shown in Fig 6B. Strand separation occurs within the DNA of the second end of the break ~3.5 kb from the I-SceI site. One possible source depicted here is a stalled replication fork. The pale orange and blue arrows flanking the stalled fork represent the exposed ssDNA sequences that template the inversion (orange) and inversion-duplication (blue) sequences identified within the LTGC breakpoint (A) The displaced nascent strand product of LTGC (black) acquires a ≥21bp insertion (red; whether templated or untemplated is unknown). (B) Microhomology-mediated base-pairing between the 3’ end of the displaced nascent strand and ssDNA of the stalled replication fork. (C) The lagging strand template enables retrograde nascent strand extension (“MMBIR”), generating the inversion sequences as shown. (D) Displacement of the nascent strand. (E) Four base pair MH-mediated (Fig 6B) annealing of the 3’ end of the displaced nascent strand with the 5’ end of the duplicated region on the leading strand. Black arrowheads: sites of endonucleolytic cleavage that would enable completion of rearrangement by MMEJ-mediated rejoining. Alternatively, more extensive MMBIR copying could complete the rearrangement.
